# Naloxone prescriptions among patients with a substance use disorder and a positive fentanyl urine drug screen presenting to the emergency department

**DOI:** 10.1186/s12954-023-00878-8

**Published:** 2023-10-05

**Authors:** Shawkut Amaan Ali, Jasmine Shell, Raymond Harris, Marshall Bedder

**Affiliations:** 1 Psychiatry and Health Behavior, Augusta University, 997 St. Sebastian Way, Augusta, GA 30912 USA; 2 Carl R. Darnall Army Medical Center, 36065 Santa Fe Ave, Fort Cavazos, TX 76544 USA

**Keywords:** Naloxone, Emergency department, Substance use disorder, Fentanyl, Harm reduction, Cocaine use, Urine drug screen, Opioid, Hospitalization, Buprenorphine

## Abstract

**Background:**

Over 109,000 people in the USA died from a drug overdose in 2022. More alarming is the amount of drug overdose deaths involving synthetic opioids other than methadone (SOOM), primarily fentanyl. From 2015 to 2020, the number of drug overdose deaths from SOOM increased 5.9-fold. SOOM are commonly being found in many other drugs without the user's knowledge. Given the alarming number of overdose deaths from illicit drugs with SOOM, naloxone should be prescribed for all persons using illicit drugs regardless of if they knowingly use opioids. How often providers prescribe naloxone for these patients remains unknown.

The aim of this study is to determine the rate of naloxone prescriptions given to patients with any substance use disorder, including when the patient has a urine drug screen positive for fentanyl. Secondary aims include determining what patient factors are associated with receiving a naloxone prescription.

**Methods:**

The design was a single-center retrospective cohort study on patients that presented to the Augusta University Medical Center emergency department between 2019 through 2021 and had an ICD-10 diagnosis of a substance use disorder. Analyses were conducted by logistic regression and *t*-test or Welch’s *t*-test.

**Results:**

A total of 10,510 emergency department visits were by 6787 patients. Naloxone was prescribed in 16.3% of visits with an opioid-related discharge diagnosis and 8.4% of visits with a non-opioid substance use-related discharge diagnosis and a urine drug screen positive for fentanyl. Patients with a fentanyl positive urine drug screen had higher odds of receiving a naloxone prescription (aOR 5.80, 95% CI 2.76–12.20, *p* < 0.001). Patients with a psychiatric diagnosis had lower odds of being prescribed naloxone (aOR 0.51, *p* = 0.03). Patients who received naloxone had a lower number of visits (mean 1.23 vs. 1.55, *p* < 0.001). Patients with a urine drug screen positive for cocaine had higher odds of frequent visits (aOR 3.07, *p* = 0.01).

**Conclusions:**

Findings should remind providers to prescribe naloxone to all patients with a substance use disorder, especially those with a positive fentanyl urine drug screen or a co-occurring psychiatric condition. Results also show that cocaine use continues to increase healthcare utilization.

**Supplementary Information:**

The online version contains supplementary material available at 10.1186/s12954-023-00878-8.

## Background

Over 109,000 people in the USA died from a drug overdose in 2022 [1]. This includes the previous 1.8-fold increase from 2015 to 2020. More alarming is the amount of drug overdose deaths involving synthetic opioids other than methadone (SOOM), primarily fentanyl. From 2015 to 2020, the number of drug overdose deaths from SOOM increased 5.9-fold. Furthermore, SOOM are commonly being found in many other drugs without the user’s knowledge [2], which is likely contributing to the significant rise in overdose deaths. From 2015 to 2020, drug overdose deaths involving prescription opioids and SOOM increased 3.8-fold, from heroin and SOOM 3.3-fold, benzodiazepines and SOOM 4.4-fold, cocaine and SOOM 9.0-fold, and psychostimulants (primarily methamphetamine) and SOOM 23.7-fold [3].

Naloxone has been well established as a safe and effective medication to reverse opioid overdose. It is now standard of care to prescribe naloxone for patients taking opioids or with an opioid use disorder (OUD). Naloxone has also been recommended by the Surgeon General and the U.S. Food and Drug Administration for anyone at-risk of opioid overdose [4, 5]. Furthermore, studies have shown take-home prescriptions of naloxone help prevent overdose deaths [6].

Given the alarming number of overdose deaths from illicit drugs with SOOM, naloxone should be prescribed for all persons using illicit drugs regardless of if they knowingly use opioids [7]. How often providers prescribe naloxone for these patients remains unknown.

Emergency department (ED) providers are often the primary and sole contact persons using drugs have with a health care provider. Therefore, it becomes critical for ED providers to prescribe naloxone when necessary.

Multiple studies have investigated the rate of naloxone prescription to patients with an OUD who visit the ED [8–12]. However, these studies included only OUD, none included patients with a non-opioid substance use disorder (SUD). It is important to include all SUDs given the common adulteration of other drugs with fentanyl [13, 14]. Furthermore, none of the studies evaluated the impact of a positive fentanyl urine drug screen (UDS) on providers likelihood of prescribing naloxone. One of the previous studies included co-occurring psychiatric diagnoses and patients with multiple SUDs, but the study did not evaluate the impact of comorbid chronic medical disease and the study was conducted only on commercially insured patients [12].

The primary objective of this study is to determine how often hospital providers prescribe naloxone to patients with any SUD, especially when the patient’s UDS is positive for fentanyl. Secondary objectives include determining if insurance status, demographics, unhoused status, co-occurring chronic medical conditions, psychiatric diagnoses, having multiple SUDs, having positive UDSs, or the presence of an addiction medicine consult service impacted the odds of being prescribed naloxone. We also aim to determine if being prescribed naloxone or any other medication for a substance use disorder (MSUD) decreased the frequency of ED visits.

## Methods

### Study design

The design was a single-center retrospective cohort study on all patients that presented to the ED between 2019 through 2021 and had an International Classification of Diseases, tenth edition (ICD-10) diagnosis of a SUD excluding patients with only nicotine use disorder as their SUD. The included ICD-10 codes were F10–F16, F18, and F19. The data were abstracted from the electronic medical records by the Augusta University’s data management team and reviewed by two of the authors, Shawkut Ali and Jasmine Shell. Ten percent of the ED visits were randomly selected for accuracy checks of the data.

### Setting

The study evaluated the medical records of patients who visited the ED of Augusta University Medical Center (AUMC). Augusta University Medical Center is a state safety-net level one trauma center serving 13 counties in East Georgia and Western South Carolina [15]. The hospital is an academic medical center that houses 50 Accreditation Council for Graduate Medical Education residencies and fellowships and is the home of Georgia’s only public medical school [16]. It is in Augusta, GA, which is the third largest city in the state with a population of 202,096. About 56.5% of the population is Black or African American alone and 22.1% of the population live in poverty [17].

### Participants

Study participants were included if they had an ICD-10 diagnosis of a SUD, excluding patients that only had nicotine use disorder as the SUD, and visited the AUMC ED between January 1, 2019, to December 31, 2021. Institutional Review Board exempt status was obtained from Augusta University.

### Variables

The main outcome variable was naloxone prescription at discharge of the ED visit or hospitalization. Other outcome variables included whether any MSUD was prescribed at discharge, a presumptive positive UDS for fentanyl, mean number of ED visits during the study time-period, and whether the patient had frequent ED visits. Medications for substance use disorders included buprenorphine (any formulation), naltrexone, methadone, acamprosate, or disulfiram. Frequent ED visits were defined by 12 visits total in the three-year span, which averages four visits per year. Four was the cut off because most ED visit frequency studies defined frequent ED usage as four visits per year [18].

Primary independent variables included age, race, gender, presence of a co-occurring psychiatric diagnosis, presence of multiple substance use disorders, a diagnosis of opioid use disorder, a chronic medical condition, unhoused status, and type of insurance. The age, race, and gender variables were based on demographic data that were entered into the electronic health record by patient self-report to registration staff. Age was further categorized into two new independent variables for analyses: less than 18 years old and 65 years old and older. Ethnicity was not used due to the significant number of inaccuracies noted by the data management team. Unhoused was defined as the patient having no place to live at the time of the ED visit. The unhoused variable was determined by manual chart review of each ED visit where the discharge diagnosis was SUD-related and there was a presumptive positive UDS for fentanyl. These specific visits were analyzed to reduce the number of visits that needed manual chart review down to a more manageable number of 405 visits. Chronic medical conditions, psychiatric and SUD diagnoses that were used in the study are listed in Additional file 1: Supplement 1.

We also included presumptive positive results of urine drug screens for fentanyl, opiates, oxycodone, amphetamines, benzodiazepines, cocaine, and tetrahydrocannabinol (THC) as independent variables. We used before and after the start of COVID and our addiction medicine inpatient consultation liaison service (AMCL) as independent variables. The year 2019 was classified as before COVID and the years 2020 and 2021 were classified as during COVID. The time between January 2019 through June 2020 was classified as before the AMCL started and from July 2020 through December 2021 as after AMCL started. The AMCL team is frequently consulted by ED providers about patients in the ED who may need addiction related care, including assessment, diagnosis, and mediation management. However, a visit made after the AMCL started did not automatically mean the team was consulted during the ED visit or hospitalization. Whether or not AMCL was consulted during the hospital stay was not determined.

### Statistical methods

All analyses were computed by the study authors using Statistical Package for the Social Sciences Software version 28.0.1.1. The university’s biostatistician advised the authors on data management issues and how to properly conduct statistical analyses. The insurance variable was missing 0.2% of the values. These values were removed from analyses and had no impact on the results. Descriptive statistics were done for all variables. Separate descriptive statistics were also done for sub-groups of the ED visits including only visits with a presumptive positive UDS for fentanyl, a current diagnosis of OUD, no current diagnosis of OUD, a discharge diagnosis SUD-related or OUD-related, and a discharge diagnosis SUD-related but excluding alcohol use disorder (AUD) and OUD. To determine if the discharge diagnosis was SUD-related we used diagnostic codes F10–F19 and T36–T50. These codes were selected based on the Centers for Disease Control and Prevention’s (CDC) guidance on overdose surveillance [19]. The ED visits were categorized into sub-groups to help determine if the rate of naloxone or MSUD prescriptions was different among the various sub-groups. This was done because we expected the rate of prescriptions to vary depending on whether the factors mentioned above were present at the ED visit.

Chi-squared or Fisher exact tests were conducted between each independent variable and outcome. The results of these tests were used to help determine any possible confounding variables for the logistic regression analyses. Logistic regression and independent samples *t*-test or Welch’s *t*-test were used for the main analyses. Logistic regression analyses were conducted on the patients’ first ED visit during the study time-period to account for multiple visits by the same patient. Statistical significance was set at a *p* value of less than 0.05.

## Results

### Study population

A total of 10,510 ED visits were made between January 1, 2019, through December 31, 2021, by 6787 patients with a SUD diagnosis, excluding patients with only nicotine use disorder. Demographics and characteristics for all ED visits and only ED visits with a presumptive positive UDS for fentanyl are shown in Table 1.

**Table 1:** Characteristics of all ED visits^a^ and ED visits^a^ with a UDS presumptive positive for fentanyl from 2019 to 2021

Variable	All ED visits^a^	ED visits^a^ with UDS fentanyl POS
	Percentage (counts)	Percentage (counts)
*Age (years)*		
Mean	45.7 (SD 15.6)	42.3 (SD 15.2)
< 18	1.6 (167/1054)	0.4 (6/1408)
18–45	48.0 (5041/10504)	61.8 (870/1408)
46–64	40.5 (4249/10504)	31.1 (438/1408)
65 and older	10.0 (1047/10504)	6.7 (94/1408)
*Sex*		
Female	31.3 (3285/10510)	33.7 (475/1408)
Male	68.7 (7225/10510)	66.3 (933/1408)
*Race*		
White	46.8 (4919/10510)	60.7 (854/1408)
Black	50.3 (5290/10510)	36.0 (507/1408)
Asian	0.3 (36/10510)	0.4 (6/1408)
Other	1.8 (187/10510)	2.0 (28/1408)
Declined to answer	0.7 (78/10510)	0.9 (13/1408)
*Insurance*		
Uninsured	39.1 (4098/10486)	46.8 (659/1405)
Private	9.9 (1036/10486)	10.2 (144/1405)
Medicaid	26.6 (2789/10486)	21.7 (305/1405)
Medicare	18.8 (1967/10486)	13.8 (194/1405)
Military	2.5 (265/10486)	1.9 (27/1405)
Other	3.2 (331/10486)	5.4 (76/1405)
Multiple substance use disorders	42.5 (4469/10510)	55.8 (786/1408)
Opioid use disorder	9.8 (1028/10510)	24.6 (346/1408)
Co-occurring psychiatric diagnosis	30.2 (3169/10510)	34.6 (487/1408)
Co-occurring medical diagnosis	45.8 (4814/10510)	39.1 (550/1408)

*UDS* urine drug screen, *ED* emergency department, *POS* positive, *SD* standard deviation

^a^All ED visits that meet study criteria

### MSUDs prescriptions

Naloxone was prescribed at 1.3% of all ED visits and 3.5% of all ED visits with a UDS presumptive positive for fentanyl. Rates of naloxone and other MSUD prescriptions for sub-groups of ED visits are found in Table 2. The odds of being prescribed any MSUD was higher after the start of AMCL, 2.66 (95% CI 1.49–4.75, *p* < 0.001).

**Table 2:** ED visits where a MSUD was prescribed at discharge from 2019 to 2021

Data Subset	Naloxone	Buprenorphine	Naltrexone	Methadone	Acamprosate	Disulfiram
	Percentage (counts)	Percentage (counts)	Percentage (counts)	Percentage (counts)	Percentage (counts)	Percentage (counts)
All ED visits^a^	1.3 (138/10510)	0.3 (33/10510)	0.6 (58/10510)	0.2 (19/10510)	0.1 (12/10510)	0.1 (9/10510)
UDS fentanyl POS	3.5 (49/1408)	1.5 (21/1408)	0.2 (3/1408)	0.2 (3/1408)	0.1 (1/1408)	0.1 (1/1408)
Current diagnosis of OUD	7.0 (72/1028)	2.6 (27/1028)	0.3 (3/1028)	0.9 (9/1028)	0.0 (0/1028)	0.1 (1/1028)
Current OUD diagnosis and UDS fentanyl POS	8.7 (30/346)	6.1 (21/346)	0.9 (3/346)	0.3 (1/346)	0.0 (0/346)	0.3 (1/346)
UDS fentanyl POS without an OUD diagnosis	1.8 (19/1062)	0.0 (0/1062)	0.0 (0/1062)	0.2 (2/1062)	0.1 (1/1062)	0.0 (0/1062)
Discharge diagnosis SUD-related	3.7 (108/2953)	0.5 (15/2953)	1.4 (42/2953)	0.1 (2/2953)	0.3 (8/2953)	0.2 (7/2953)
Discharge diagnosis OUD-related	16.3 (50/307)	2.6 (8/307)	0.3 (1/307)	0.3 (1/307)	0.0 (0/307)	0.0 (0/307)
Discharge diagnosis SUD-related and UDS fentanyl POS	9.1 (37/405)	2.0 (8/405)	0.5 (2/405)	0.2 (1/405)	0.0 (0/405)	0.2 (1/405)
Discharge diagnosis SUD-related excluding OUD and AUD	4.9 (51/1038)	0.3 (3/1038)	0.3 (1038)	0.0 (0/1038)	0.0 (0/1038)	0.0 (0/1038)
Discharge diagnosis SUD-related excluding OUD and AUD but with a UDS fentanyl POS	8.4 (19/225)	0.0 (0/225)	0.9 (2/225)	0.0 (0/225)	0.0 (0/225)	0.0 (0/225)

*ED* emergency department, *MSUD* medication for a substance use disorder, *UDS* urine drug screen, *OUD* opioid use disorder, *SUD* substance use disorder, *AUD* alcohol use disorder, *POS* positive

^a^All ED visits that meet study criteria

### Odds of naloxone prescription

Odds of being prescribed naloxone when there was a presumptive positive UDS for fentanyl was 5.80 (95% CI 2.76–12.20, *p* < 0.001) after adjusting for White race, current OUD diagnosis, and a presumptive positive UDS for opiates or oxycodone. Among those without a current OUD diagnosis, the odds of being prescribed naloxone when there was a presumptive positive UDS for fentanyl was 7.09 (95% CI 2.48–20.25, *p* < 0.001) after adjusting for presence of White race, and a presumptive positive UDS for opiates or oxycodone. Odds of naloxone being prescribed based on various other characteristics are found in Table 3.

**Table 3:** Odds of being prescribed naloxone at discharge of first ED visit by various characteristics. Notable results

Variable	Data Subset	OR	95% CI of OR	*p* Value	Covariate (s)	aOR^a^	95% CI of aOR	*p* Value
AMCL started	All Patients	1.547	1.089–2.197	0.02*	UDS fentanyl POS	1.259	0.751–2.109	0.38
During COVID^b^	All Patients	2.132	1.360–3.343	< 0.001*	AMCL started; UDS fentanyl POS	2.325	1.006–5.371	0.05*
UDS fentanyl POS	All Patients	12.056	6.636–21.901	< 0.001*	UDS oxy POS; UDS opiates POS; Patient race-White; OUD dx	5.800	2.758–12.198	< 0.001*
UDS fentanyl POS	OUD dx	2.947	1.099–7.902	0.03*	UDS oxy POS; UDS opiates POS; Patient race-White	3.259	1.199–8.858	0.02*
UDS fentanyl POS	No OUD dx	11.493	4.164–31.722	< 0.001*	UDS oxy POS; UDS opiates POS; Patient race-White	7.089	2.481–20.252	< 0.001*
65yo or older	All Patients	0.152	0.037–0.617	0.008*	UDS fentanyl POS; UDS oxy POS; UDS opiates POS	0.212	0.029–1.557	0.13
OUD dx	All Patients	11.843	8.016–17.496	< 0.001*	UDS fentanyl POS; UDS oxy POS; UDS opiates POS	5.482	2.885–10.418	< 0.001*
Co-occurring Psych dx	All Patients	0.300	0.178–0.506	< 0.001*	UDS fentanyl POS	0.513	0.281–0.935	0.03*
Co-occurring chronic medical condition	All Patients	0.245	0.158–0.382	< 0.001*	65yo or older; OUD dx; UDS fentanyl POS	0.629	0.357–1.106	0.11
Uninsured	All Patients	2.532	1.792–3.576	< 0.001*	UDS fentanyl POS	1.271	0.769–2.100	0.35
Medicaid	All Patients	0.410	0.249–0.674	< 0.001*	UDS fentanyl POS	0.693	0.359–1.339	0.28
Medicare	All Patients	0.447	0.252–0.793	0.006*	UDS fentanyl POS	0.603	0.258–1.409	0.24
Private insurance	All Patients	0.884	0.483–1.619	0.69				
Patient race-White	All Patients	3.157	2.153–4.630	< 0.001*	UDS fentanyl POS; OUD dx	1.278	0.723–2.261	0.40
Patient race-Black	All Patients	0.283	0.189–0.423	< 0.001*	UDS fentanyl POS; OUD dx	0.669	0.368–1.217	0.19
Patient race-Black	OUD dx	0.832	0.473–1.464	0.52				
Patient race-White	OUD dx	0.995	0.545–1.815	0.99				
UDS amp POS	All Patients	2.685	1.522–4.763	< 0.001*	UDS fentanyl pos	1.254	0.693–2.269	0.45
UDS fentanyl POS	UDS amp POS	4.860	1.632–14.469	0.005*	OUD dx	3.388	1.091–10.522	0.04*
UDS benzo POS	All Patients	1.766	0.931–3.347	0.08				
UDS fentanyl POS	UDS benzo POS	13.401	1.733–103.640	0.01*	OUD dx	9.425	1.178–75.440	0.04*
UDS cocaine POS	All Patients	1.218	0.632–2.345	0.56				
UDS fentanyl POS	UDS cocaine POS	6.899	2.055–23.163	0.002*	OUD dx	2.519	0.644–9.852	0.18
UDS opiates POS	All Patients	5.606	3.179–9.883	< 0.001*	UDS fentanyl pos	3.135	1.740–5.647	< 0.001*
UDS fentanyl POS	UDS opiates POS	4.767	1.776–12.793	0.002*	OUD dx	3.687	1.341–10.141	0.01*
UDS oxy POS	All Patients	6.876	3.803–12.429	< 0.001*	UDS fentanyl pos	4.385	2.385–8.060	< 0.001*
Unhoused^c^	Discharge dx SUD and UDS fentanyl POS	0.685	0.202–2.328	0.54				
UDS amp POS	UDS Fentanyl POS	1.008	0.530–1.915	0.98				
UDS opiates	UDS Fentanyl POS	2.506	1.316–4.770	0.005*	OUD dx	1.570	0.791–3.118	0.20
UDS oxy POS	UDS Fentanyl POS	3.126	1.568–6.233	0.001*	OUD dx	2.425	1.192–4.934	0.01*

^a^Adjusted odds ratio noted when covariate(s) is/are used

^b^During COVID was defined as years 2021 and 2020 and before COVID as year 2019

^c^Unhoused was defined as the patient having no place to live at the time of the ED visit

* statistically significant,* p* < 0.05

Reference groups for each variable

AMCL started—visits prior to AMCL starting; During COVID—visits during 2019; UDS fentanyl POS—UDS fentanyl negative; 65yo or older—less than 65 years old; OUD dx—no OUD dx; Co-occurring psych dx—no psych dx; Co-occurring chronic medical condition—no chronic medical condition; Uninsured—all other insurances; Medicaid—all other insurances; Medicare—all other insurances; Private insurance—all other insurances; Patient race-White—all other races; Patient race-Black—all other races; UDS amph POS—UDS amph negative; UDS benzo POS—UDS benzo negative; UDS cocaine POS—UDS cocaine negative; UDS opiate POS—UDS opiate negative; UDS oxy POS—UDS oxy negative; Unhoused—not unhoused

ED—emergency department; Psych—psychiatric; UDS—urine drug screen; AMCL—addiction medicine inpatient consult liaison service; OUD—opioid use disorder; yo—years old; POS—positive; dx—diagnosis; oxy—oxycodone; benzo-—benzodiazepine; SUD—substance use disorder; THC—tetrahydrocannabinol

### Odds of having a UDS fentanyl positive

The odds of having a UDS presumptive positive for fentanyl were lower for Black patients (adjusted odds ratio, aOR 0.60, 95% CI 0.51–0.70, *p* < 0.001) and higher for White patients (aOR 1.63, 95% CI 1.41–1.91, *p* < 0.001). The odds were also higher for patients who had a UDS presumptive positive for amphetamines (aOR 4.46, 95% CI 3.77–5.27, *p* < 0.001), benzodiazepines (aOR 3.54, 95% CI 2.93–4.27, *p* < 0.001), and opiates (aOR 2.97, 95% CI 2.41–3.67, *p* < 0.001). Other notable findings can be found in Table 4.

**Table 4:** Odds of having a UDS presumptive positive for fentanyl at first ED Visit during the study time-period 2019–2021. Notable results

Variable	Data Subset	OR	95% OR CI	*p* Value	Covariate (s)	aOR^a^	95% aOR CI	*p* Value
During COVID^b^	All Patients	1.855	1.580–2.177	< 0.001*				
Multiple SUDs	All Patients	1.597	1.384–1.842	< 0.001*	OUD dx	1.355	1.166–1.574	< 0.001*
Co-occurring Psych dx	All Patients	0.839	0.719–0.978	0.03*				
Co-occurring medical dx	All Patients	0.771	0.667–0.891	< 0.001*	Age 65 and older; OUD dx	0.819	0.700–0.957	0.01*
< 18 years old	All Patients	0.165	0.072–0.377	< 0.001*	OUD diagnosis	0.187	0.081–0.431	< 0.001*
Age 65 and older	All Patients	0.683	0.532–0.878	0.003*	OUD dx; Co-occurring medical dx	0.742	0.567–0.971	0.03*
Uninsured	All Patients	1.323	1.146–1.526	< 0.001*	Patient race – White; OUD dx	1.251	1.075–1.456	0.004*
Medicaid	All Patients	0.792	0.663–0.945	0.01*	Patient race-Black	0.868	0.724–1.040	0.13
Medicare	All Patients	0.758	0.618–09.29	0.008*	Age 65 and older	0.861	0.678–1.093	0.22
Patient race-Black	All Patients	0.499	0.430–0.579	< 0.001*	Medicaid; OUD dx	0.600	0.513–0.701	< 0.001*
Patient race-White	All Patients	1.950	1.684–2.257	< 0.001*	Uninsured; OUD dx	1.638	1.405–1.909	< 0.001*
OUD dx	All Patients	6.539	5.251–8.142	< 0.001*	Patient race-White	5.789	4.633–7.234	< 0.001*
UDS amph POS	All Patients	4.406	3.767–5.153	< 0.001*	OUD dx; Multiple SUDs; UDS benzo, oxy, opiates POS	4.456	3.767–5.270	< 0.001*
UDS benzo POS	All Patients	3.594	3.027–4.268	< 0.001*	OUD dx; Multiple SUDs; UDS amph, oxy, opiates POS	3.540	2.933–4.273	< 0.001*
UDS cocaine POS	All Patients	0.867	0.726–1.036	0.12				
UDS opiate POS	All Patients	3.608	3.040–4.282	< 0.001*	OUD dx; Multiple SUDs; UDS amph, benzo, oxy POS	2.973	2.410–3.668	< 0.001*
UDS oxy POS	All Patients	2.690	2.136–3.386	< 0.001*	OUD diagnosis; Multiple SUDs; UDS amph, benzo, opiates POS	1.181	0.884–1.578	0.26
UDS THC POS	All Patients	1.294	1.116–1.499	< 0.001*	OUD dx; Multiple SUDs; UDS amph POS	1.164	0.989–1.369	0.07

^a^Odds ratio adjusted by covariates listed

^b^During COVID was defined as years 2021 and 2020 and before COVID as year 2019

* statistically significant,* p* < 0.05

Reference groups for each variable

Multiple SUDs—only one SUD; During COVID—visits during 2019; UDS fentanyl POS—UDS fentanyl negative; < 18 years old—18yo and older; 65yo or older—less than 65 years old; OUD dx—no OUD dx; Co-occurring psych dx—no psych dx; Co-occurring chronic medical condition—no chronic medical condition; Uninsured—all other insurances; Medicaid—all other insurances; Medicare—all other insurances; Private insurance—all other insurances; Patient race-White—all other races; Patient race-Black—all other races; UDS amph POS—UDS amph negative; UDS benzo POS—UDS benzo negative; UDS cocaine POS—UDS cocaine negative; UDS opiate POS—UDS opiate negative; UDS THC POS—UDS THC negative; UDS oxy POS—UDS oxy negative

*ED*—emergency department; UDS—urine drug screen; AMCL—addiction medicine consult liaison service; Psych—psychiatric; OUD—opioid use disorder; POS—positive; dx—diagnosis; amph—amphetamine; benzo—benzodiazepine; oxy—oxycodone

### ED Visit frequency

The mean number of ED visits was lower for those prescribed naloxone at their first ED visit, 1.23 versus 1.55 (mean difference 0.32, 95% CI 0.21–0.43, *p* < 0.001). Patients with a presumptive positive UDS for fentanyl at their first ED visit had a lower mean number of ED visits, 1.39 versus 1.63 (mean difference 0.24, 95% CI 0.14–0.33, *p* < 0.001). There was no statistically significant difference in mean number of ED visits by whether a patient was prescribed any MSUD. Other notable findings can be found in Additional file 1: Supplement 2.

Odds of frequent ED visits was lower for female patients, 0.38 (95% CI 0.16–0.90, *p* = 0.03). Odds of frequent ED visits were higher for patients with a presumptive positive UDS for cocaine, 3.069 (95% CI 1.30–7.24, *p* = 0.01) after adjusting for Black race and Medicaid insurance. Notable results are presented in Table 5.

**Table 5:** Odds of frequent ED visits. Notable results

Variable	OR	95% CI	*p* Value	Covariate(s)	aOR^a^	95% CI	*p* Value
Medicaid	3.807	2.026–7.152	< 0.001*	Patient race-Black; UDS cocaine POS	2.448	1.084–5.533	0.03*
Patient Race-Black	2.422	1.225–4.789	0.01*	Medicaid; UDS cocaine POS	2.561	0.890–7.367	0.08
Patient Race-White	0.457	0.231–0.904	0.02				
Sex-Female	0.378	0.158–0.903	0.03*				
UDS cocaine POS	4.478	1.999–10.032	< 0.001*	Patient race-Black; Medicaid	3.069	1.300–7.244	0.01*
UDS fentanyl POS	0.423	0.126–1.421	0.16				
Multiple SUDs	1.164	0.617–2.195	0.64				
Co-occurring Psych dx	1.068	0.530–2.149	0.85				

Frequent ED visits were defined as 12 visits over the 3-year period (average of 4 visits per year)

^a^Odds ratio adjusted for selected covariates

* statistically significant,* p* < 0.05

Reference groups for each variable

Medicaid—all other insurances; Patient Race-Black—all other races; Patient Race-White—all other races; Sex-Female—male; UDS cocaine POS—UDS cocaine negative; UDS fentanyl POS—UDS fentanyl negative; Multiple SUDs—only one SUD; Co-occurring psych dx—no psych dx

*ED* emergency department, *UDS* urine drug screen, *POS* positive, *psych* psychiatric, *dx* diagnosis, *Psych* psychiatric

## Discussion

This study represented the first, to our knowledge, to examine differences in hospital provider naloxone prescribing to patients presenting to a state-funded hospital with any SUD, with or without a positive UDS for fentanyl. Overall, naloxone prescribing was very low for all sub-groups. Even among those with a discharge diagnosis OUD-related the rate was only 16.3%. For those with a current diagnosis of OUD and a presumptive positive UDS for fentanyl the rate was 8.7%. These rates are lower than previous studies [8–12], despite our study including patients that also had a presumptive positive UDS for fentanyl. The rates were also lower despite our study including visits that occurred after the expansion of the CDC’s overdose awareness campaign [20] in 2019 and the FDA’s recommendation for naloxone that was released in July 2020 [4].

We believe these low rates may be due to a need for more provider education in addiction and increased stigma in our region compared to other areas of the USA, especially compared to large metropolitan cities. Evidence that the lack of provider education is contributing to the lower rates may be evident by the finding that the start of the AMCL service increased the odds of being prescribed any MSUD, (OR 2.66, *p* < 0.001). However, there was no statistically significant increase in naloxone prescribing after the start of AMCL.

Most notable was that having a presumptive positive UDS for fentanyl significantly increased the odds of being prescribed naloxone (aOR 5.80, *p* < 0.001). This was expected as providers are increasingly being made aware of the dangers of fentanyl and overdose.

Among patients that did not have an OUD, those with a presumptive positive UDS for fentanyl had significantly higher odds of receiving naloxone (aOR 7.09, *p* < 0.001), which was higher odds than those with an OUD (aOR 3.26, *p* = 0.02). We believe this is likely due to a limitation in our study. Patients with a current OUD are more likely to already have naloxone from a previous provider and they may have informed the discharging provider that they already have naloxone.

Patients with a psychiatric diagnosis had significantly lower odds of being prescribed naloxone (aOR 0.51, *p* = 0.03). This may be because the psychiatric complaints, such as suicidal ideation, are so heavily focused during the hospital stay that the substance use component is secondary. It is critical that providers remember to also address substance use in patients with psychiatric diagnoses, as they are at even higher risk for overdose [21].

Those who were prescribed naloxone at their first ED visit had a lower mean number of ED visits (mean difference, 0.32, *p* < 0.001). Additionally, no patients that were prescribed naloxone or any MSUD at their first ED visit had frequent ED visits. This reinforces previous research on naloxone decreasing ED visit frequency [22]. Those who had a presumptive positive UDS for fentanyl at their first ED visit had a lower mean number of ED visits. We believe this is likely because those patients were more likely to be prescribed naloxone after providers noticed their UDS results, which could have led to a lower number of future ED visits.

Previous studies have found disparities in naloxone prescribing among patients of color [8–12]. Initially, our findings were similar, but after adjusting for confounding variables this difference was no longer statistically significant. We found that White patients were more likely to have a presumptive positive UDS for fentanyl (aOR 1.64, *p* < 0.001) and a current OUD diagnosis (OR 3.03, *p* < 0.001) and Black patients less likely (aOR 0.60, *p* < 0.001 and OR 0.32, *p* < 0.001, respectively). After adjusting for these confounders, the odds of being prescribed naloxone was still higher for White patients (1.28, *p* = 0.40) and lower for Black patients (0.67, *p* = 0.18), but they were no longer statistically significant. In our population, it is possible that at least part of this racial disparity may be accounted for by the finding that White patients were more likely to have a presumptive positive UDS for fentanyl and a current OUD diagnosis, which led to higher odds of being prescribed naloxone. While not statistically significantly noted in naloxone prescriptions, racial disparities remained in other areas for our study. White patients had higher odds of being prescribed any MSUD, 2.962 (95% CI 1.39–6.32, *p* = 0.005) and Black patients lower odds, 0.28 (95% CI 0.12–0.63, *p* = 0.002) even after adjusting for current OUD diagnosis and having a presumptive positive UDS for fentanyl. Our complete findings reinforce the existence of racial disparities in substance use treatment, but the significance of having a presumptive positive UDS for fentanyl and a current OUD also are important to consider when analyzing data by race.

Another significant finding in our population was that those with a presumptive positive UDS for amphetamines, benzodiazepines, or opiates had increased odds of a presumptive positive UDS for fentanyl, (aORs) 4.46, 3.54, 2.97, respectively. This may help inform providers in similar regions or patient populations to know which substances are most likely adulterated with fentanyl in their area. Furthermore, the patients with a presumptive positive UDS for amphetamines, benzodiazepines, or opiates, had higher odds of being prescribed naloxone if they also had a presumptive positive UDS for fentanyl, (aORs) 3.39, 9.43, 3.69, respectively. This highlights how providers realized the importance of prescribing naloxone when the patient was using multiple substances.

Patients with a presumptive positive UDS for cocaine had the highest odds of frequent ED visits (aOR 3.07, *p* = 0.01), even when controlling for possible confounders, Black race, and Medicaid insurance. This is likely due to the often-forgotten complications of cocaine use and the lack of any proven medications to help decrease use, overdoses, and mortality.

## Limitations

This study had several limitations. One limitation is that we did not account for the possibility that patients may have already had naloxone when assessing whether it was prescribed by the hospital provider. During the accuracy checks of the data, ED visits were examined to see if the patient already had naloxone on their medication list, and less than 10 visits had this problem. However, it is possible that patients received naloxone from sources outside our healthcare system, such as a non-profit harm reduction organization, and therefore the prescription was not reflected in our electronic medical records. Nevertheless, given the increasing number of naloxone that needs to be administered during an overdose [23], providers may determine certain patients are at high-risk for a repeat overdose that may require multiple naloxone administrations. This may prompt providers to prescribe multiple naloxone kits to increase the chances of reversing an overdose [23].

Our study had a low number of pediatric ED visits. We believe this is because of a limitation in the inclusion criteria for the study. We included only patients who had a diagnosis of a SUD. It is possible that many pediatric patients who misuse substances did not yet have a diagnosis of a SUD and therefore were not included in the study.

False positive results on UDSs are another limitation for this study. Only presumptive positive results were analyzed. It is also possible that UDS results were appropriately positive because patients were appropriately prescribed controlled substances, such as medications for attention-deficit hyperactivity disorder. Nevertheless, in ED there is rarely time to get confirmatory testing. Clinical decisions must be made with just a UDS. However, a presumptive positive result, at least, gives providers an opportunity to start a conversation about the result.

History of COVID was included as a chronic medical condition to attempt to capture patients who may have developed a post-COVID-19 condition. Providers were unable to use the correct post-COVID-19 condition code for most of the study time period because it was not created until near the end of 2021.

As with all chart review research, electronic medical records commonly have errors or missing information. This was evident in the decision to forego ethnicity as a variable, which limited the accuracy of our race variable. There were also no available data regarding non-binary gender as a variable. We tried to alleviate these limitations by performing random accuracy checks of the data.

## Conclusions

In our study, naloxone prescription rates for patients with any SUD (with or without an OUD) who presented to the ED were very low, even when a presumptive positive UDS for fentanyl was found. However, the presence of this positive result significantly increased the odds of being prescribed naloxone. Our study results indicate that naloxone prescribing remains low at our hospital despite new recommendations from the FDA and CDC [5, 20]. Future studies should be done to confirm our results and address the study limitations.

Similarly, we also found low rates of prescribing buprenorphine or any MSUD. Patients with a current OUD and a presumptive positive UDS for fentanyl were prescribed buprenorphine at only 6.1% of the visits. This represents a missed opportunity and reiterates the importance of consulting an addiction medicine consult team and developing bridge programs to help facilitate starting patients on MSUDs.

Racial disparities continue to exist in substance use treatment, but the significance of having a presumptive positive UDS for fentanyl is also important to consider as a possible confounder when analyzing data by race.

Cocaine use continues to contribute to frequent ED visits, likely due to no FDA-approved medications to treat the disorder or prevent complications including overdose. More research is needed for stimulant use disorder-cocaine type, not only for the patient’s health, but also because the disorder increases health care utilization and costs.

With the recent ruling that naloxone will be available over the counter, there is concern that providers may be less inclined to prescribe it, which could create a new barrier for patients if it is not affordable. Future funding should be allocated to educational programs for all providers on the importance of prescribing naloxone to all patients with any SUD, especially those with a positive UDS for fentanyl. These programs should include informing providers on the importance of prescribing multiple naloxone prescriptions, training on stigma including race-related stigma, and harm reduction.

### Supplementary Information

**Additional file 1:** Diagnoses included in the study and differences in mean number of ED visits by selected variables.

## Data Availability

The dataset generated and analyzed during the current study are not publicly available due to an inability to maintain patient confidentiality for some of the patients. Specific datasets may be available from the corresponding author on reasonable request.

## Abbreviations

SOOM: Synthetic opioids other than methadone
OUD: Opioid use disorder
ED: Emergency department
SUD: Substance use disorder
UDS: Urine drug screen
MSUD: Medication for a substance use disorder
ICD-10: International Classification of Diseases, tenth edition
AUMC: Augusta University Medical Center
THC: Tetrahydrocannabinol: COVID: Coronavirus disease of 2019
AMCL: Addiction medicine inpatient consultation liaison service
AUD: Alcohol use disorder
CDC: Centers for Disease Control and Prevention
aOR: Adjusted odds ratio
