# Supplementary material for: Naloxone prescriptions among patients with a substance use disorder and a positive fentanyl urine drug screen presenting to the emergency department
Source: Harm Reduct J. 2023 Oct 5;20:144. doi: 10.1186/s12954-023-00878-8 (PMC10552257; doi:10.1186/s12954-023-00878-8)
Supplement: Supplementary file 1 — Additional file 1: Diagnoses included in the study and differences in mean number of ED visits by selected variables. [file 12954_2023_878_MOESM1_ESM.docx]

| **Supplement 1** | | |
| --- | --- | --- |
| Diagnoses included in the study | | |
| Substance use disorders | Psychiatric diagnoses | Medical diagnoses |
|  |  |  |
| F10– alcohol use disorders | Major depressive disorder | Hyperlipidemia |
| F11- opioid use disorders | All unspecified depressive disorders | Hypertension |
| F12- cannabis use disorders | Generalized anxiety disorder | Osteoarthritis |
| F13- sedative, hypnotic, anxiolytic use disorders | Panic disorder | Rheumatoid arthritis |
| F14- cocaine use disorders | All unspecified anxiety disorders | Cancer, any type |
| F15- other stimulant use disorders | Anorexia Nervosa | Heart failure, any type |
| F16- hallucinogen use disorders | Attention deficit hyperactive disorder | Coronary artery disease |
| F17- nicotine use disorders | Antisocial personality disorder | Cerebrovascular disease |
| F18- inhalant use disorders | Avoidant personality disorder | Asthma |
| F19- other psychoactive substance use disorders | Binge eating disorder | Chronic Obstructive Pulmonary Disease |
|  | Bipolar disorder | Chronic kidney disease or end-stage renal disease |
|  | All unspecified mood disorders | Diabetes type 1 and 2 |
|  | Borderline personality disorder | Dementia, any type |
|  | Bulimia nervosa | Obesity |
|  | Dependent personality disorder | Atrial fibrillation |
|  | Histrionic personality disorder | COVID-19, history or long COVID |
|  | Narcissistic personality disorder | Chronic hepatitis, cirrhosis |
|  | Obsessive compulsive disorder | Hepatitis B and C |
|  | Obsessive compulsive personality disorder | HIV |
|  | Paranoid personality disorder |  |
|  | Schizophrenia |  |
|  | Schizoaffective disorder |  |
|  | All unspecified psychotic disorders |  |
|  | Schizoid personality disorder |  |
|  | Schizotypal personality disorder |  |

| **Supplement 2** | | | | |
| --- | --- | --- | --- | --- |
| Mean difference of ED visits by selected variables present at first ED visit | | | | |
| **Variable** | **Mean (SD)** | **Mean Difference** | **95% CI of Mean Diff** | **p Value** |
|  |  |  |  |  |
| Naloxone prescribed at first ED visit | |  |  |  |
| Yes | 1.23 (0.542) | 0.320 | 0.208 to 0.432 | **<0.001*** |
| No | 1.55 (1.796) | | | |
|  |  |  |  |  |
| Any MSUD prescribed at first ED visit | | | | |
| Yes | 1.77 (1.918) | - 0.225 | -0.90 to 0.240 | 0.34 |
| No | 1.55 (1.782) | | | |
|  |  |  |  |  |
| UDS Fentanyl POS | | | | |
| Yes | 1.39 (1.163) | 0.236 | 0.140 to 0.333 | **<0.001*** |
| No | 1.63 (1.824) | | | |
|  |  | | | |
| Multiple SUDs | | | | |
| Yes | 1.65 | -0.174 | -0.265 to -0.084 | **<0.001*** |
| No | 1.48 | | | |
|  |  |  |  |  |
| Co-occurring Psych dx | | | | |
| Yes | 1.65 | -0.137 | -0.231 to -0.042 | **0.005*** |
| No | 1.51 | | | |

Analyzed by independent sample t-test or Welch’s t-test

ED- emergency department; UDS- urine drug screen; MSUD-medication for substance use disorder; POS- positive; dx- diagnosis; Psych- psychiatric
